# In the human, true myocutaneous junctions of skeletal muscle fibers are limited to the face

**DOI:** 10.1111/joa.13419

**Published:** 2021-02-27

**Authors:** Christian Albrecht May, Silvia Bramke

**Affiliations:** ^1^ Department of Anatomy Medical Faculty Carl Gustav Carus TU Dresden Dresden Germany

**Keywords:** insertion, mimic muscles, skin, striated muscle

## Abstract

Within the panniculus carnosus‐associated skeletal muscles in the human, the palmaris brevis and the platysma showed myotendinous/myofascial junctions with clear distance to the corium and the specific connection collagen type XXII. The orbicularis oris muscle, in contrast, contained bundles of striated muscle fibers reaching the corium at two distinct levels: the predominant inner ending was connected to the elastic network of the inner corium and the outer ending was within the more superficial collagen network. At both locations, the striated muscle fibers showed brush‐like cytoplasmic protrusions connecting a network which was not oriented toward the muscle fibers. Collagen type XXII was not present.

## Graphical abstract

While palmaris brevis and platysma muscles show true myotendinous junctions, the orbicularis oris muscle develops mimic muscle‐specific myocutaneous junctions. They insert brush like into the dermis and do not contain collagen XXII.

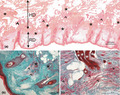

## 1 INTRODUCTION

The majority of skeletal muscle fibers are covered by a muscle fascia that separates individual or groups of muscles from the skin. The individual fiber terminations contact either the inner aspect of this fascia (myofascial junctions) or form myotendinous junctions at the tendons as intermediates to the final osseous insertions.

At the ultrastructural level, myotendinous and presumably, albeit not specifically studied, myofascial junctions are characterized by numerous finger‐like cytoplasmic processes and lateral grooves of the muscle fibers connecting intensely with ridge‐like protrusions of the tendon (Knudsen et al., 2015). This intense contact area develops from simply conical or brush‐like evaginations at birth (Mair & Tomé, 1972; Nagano et al., 1998; Ovalle, 1987) and increases further during training (Curzi et al., 2015, 2016; Kojima et al., 2008; Rissatto Sierra et al., 2018), while immobilization (Kannus et al., 1992) or loss of gravity (Carnino et al., 2000; Tidball & Quan, 1985; Zamora et al., 1995) decreases the contact area. The myotendinous junction remains to be a highly active interface (Jakobsen et al., 2018) leading to specific age‐related changes in rodents including thickening of extracellular material and increasing of the contact area (Ciena et al., 2010, 2012; Nielsen et al.,, 2018). Numerous cellular molecules are described at the myotendinous junction, like dystrophin (Samitt & Bonilla, 1990; Zhao et al., 1992), nestin (Vaittinen et al., 1999), laminin receptor integrin alpha7beta1 (Bao et al., 1993; Miosge et al., 1999; Welser et al., 2009), talin (Bozyczko et al., 1989; Tidball et al., 1986), vinculin (Law et al., 1994), and desmin (Tidball, 1992). Specific extracellular structures located at the myotendinous junction include laminin alpha1 (Pedrosa‐Domellöf et al., 2000), thrombospondin (Subramanian et al., 2007), and collagen type XXII (Jakobsen et al., 2017).

A small number of skeletal muscles, however, are named to insert directly in the dermal or subcutaneous layer of the skin. They are summarized as panniculus carnosus muscle and include various muscles of the face and trunk (reviewed by Naldaiz‐Gastesi et al., 2018). While myotendinous junctions have been studied in some detail, almost no information exists about the morphology of myocutaneous junctions. To clarify this specific connection we studied the orbicularis oris, palmaris brevis, and platysma muscle in the human.

## 2 MATERIAL AND METHODS

### 2.1 Tissue preparation

Muscle specimens of the orbicularis oris, palmaris brevis, and platysma muscle were collected from 11 human cadavers. They were part of the donor program of the Department of Anatomy in Dresden (Germany) and had given in their lifetime a written consent that their body might be used in purpose of science and education after death. There were 6 male and 5 female cadavers, age range was between 70 and 96 years, lacking neuro‐ or myopathies in their medical history as far as documented. All cadavers were fixed 2–4 days post mortem with a mixture of formalin and alcohol and remained in that solution for at least 1 year. Regions of suspected myocutaneous junctions were identified and small blocks (edge length about 1.5 cm) of the skin and underlying tissue were excised. The samples were washed several times in phosphate buffered saline (PBS, pH 7.4, 0.01 M) and then processed for embedding in paraffin wax.

### 2.2 Histology and immunohistochemistry

Serial sections (5 μm thick) of each specimen were performed in different planes and selected sections stained with hematoxylin and eosin (H&E), Goldner trichrome, or Sirius red solution.

For immunohistochemistry, consecutive sections of H&E stained sections containing myocutaneous junctions were dewaxed, rehydrated, and irradiated with microwaves in 0.01 M sodium citrate buffer (pH 6.0) for 2 × 5 min at 800 W to unmask the antigens. After washing in PBS, the sections were treated with 0.3% hydrogen peroxide for 10 min and blocked in normal mouse serum for 15 min at 37°C followed by washing in PBS. The primary polyclonal rabbit antibody against Collagen XXIIA1 (aa 181–273; Creative Diagnostics; dilution 1:100) was incubated over night at 4°C. After washing in PBS, an appropriate biotinylated secondary antibody was added and incubated for 15 min at 37°C, followed by washing and incubation with a VECTASTAIN^®^ Elite ABC mouse kit (PK 6101, PK 6102 Vector Laboratories Inc.). Visualization of peroxidase activity was realized by adding 3,3‐diaminobenzidine for 8 min.

The sections were examined on Zeiss Jenamed2 microscope (Carl Zeiss AG) and images were recorded by using a Digital Sight DS‐Fi1 camera (Nikon AG).

### 2.3 Semi‐thin sections

From selected regions of the orbicularis oris muscle small specimens (3 × 3 × 3 mm) were post‐fixed in glutaraldehyde and embedded in Epon. Serial semi‐thin sections were performed and stained with toluidine blue to reconstruct the myocutaneous junction in more detail.

## 3 RESULTS

**FIGURE 1:**
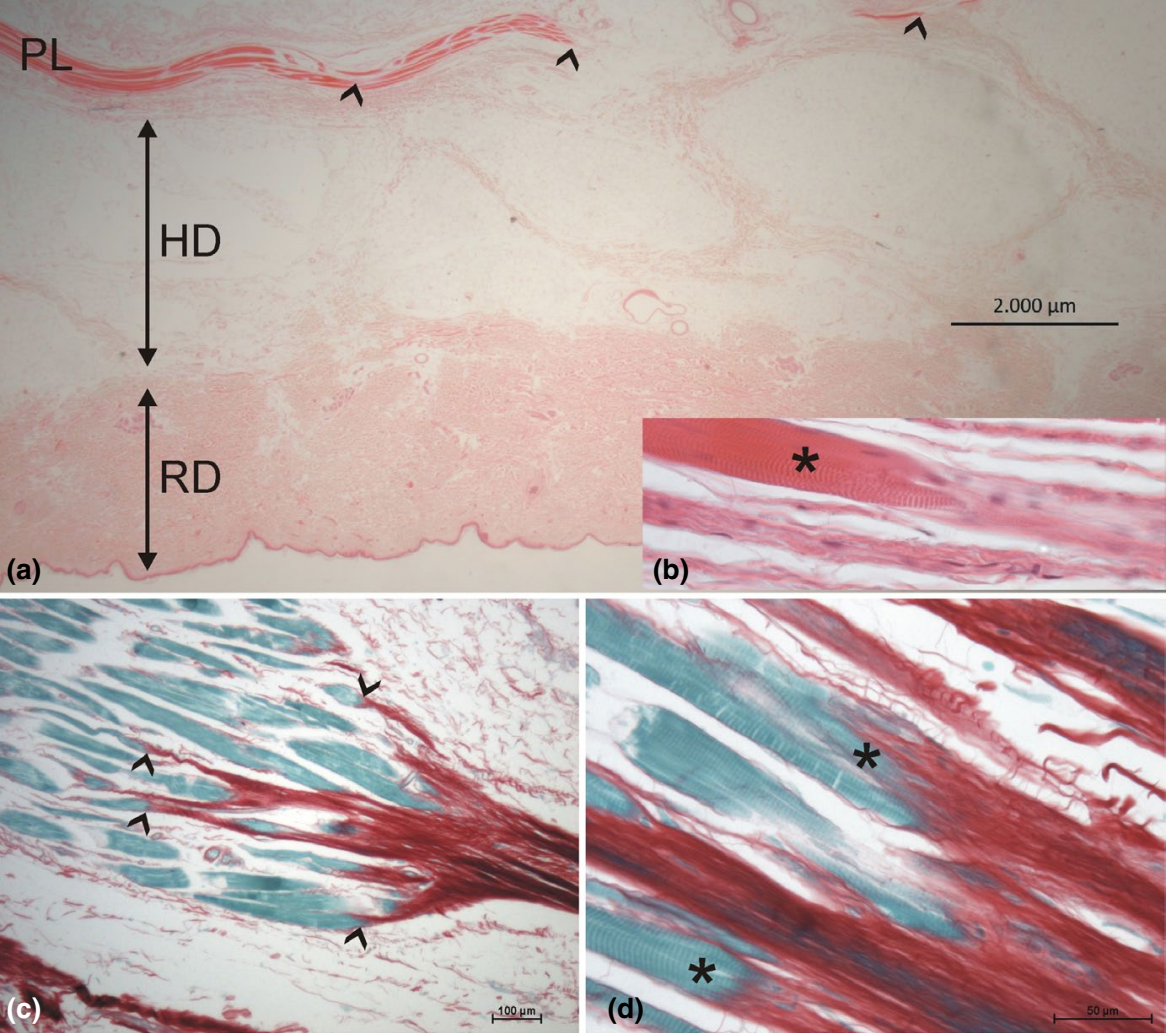
(a,b) HE staining of the platysma (PL) and its connection to the skin. A. Note the location of the platysma in the hypodermis (HD) with notable distance to the reticular dermis (RD). Myotendinous junctions (shown in b; the muscle fiber marked by an asterisks) are marked with arrowheads. Note the subcutaneous retinacula (collagen fiber strands) connecting the muscle with the reticular dermis. (c,d) Sirius red staining of the myotendinous junction of the palmaris brevis muscle. (c) Note the clear endings of the individual muscle fibers (green) at collagen fiber bundles (red), marked by arrowheads. (b) Higher magnification shows the parallel finger‐like protrusions of the muscle fibers (asterisks) toward the tendon‐like dense collagen fibers

Serial sections through the palmaris brevis and platysma muscle revealed that both muscles do generally not form true myocutaneous junctions. Instead, they insert into connective tissue fasciae of either the hypothenar muscles (in case of the palmaris brevis muscle) or the superficial body fascia (in case of the platysma; Figure 1a). These insertions show myotendinous junctions and formation of multiple small tendons connecting the individual muscle fibers and the corresponding fascia (Figure 1b–d). The muscle fibers always ended in a clear distance to the dermis. The fascia of the muscles connected to the dermis via subcutaneous retinacula (Figure 1a). Myocutaneous junctions were therefore limited in this study to the orbicularis oris muscle.

### 3.1 Myocutaneous junctions of the orbicularis oris muscle

**FIGURE 2:**
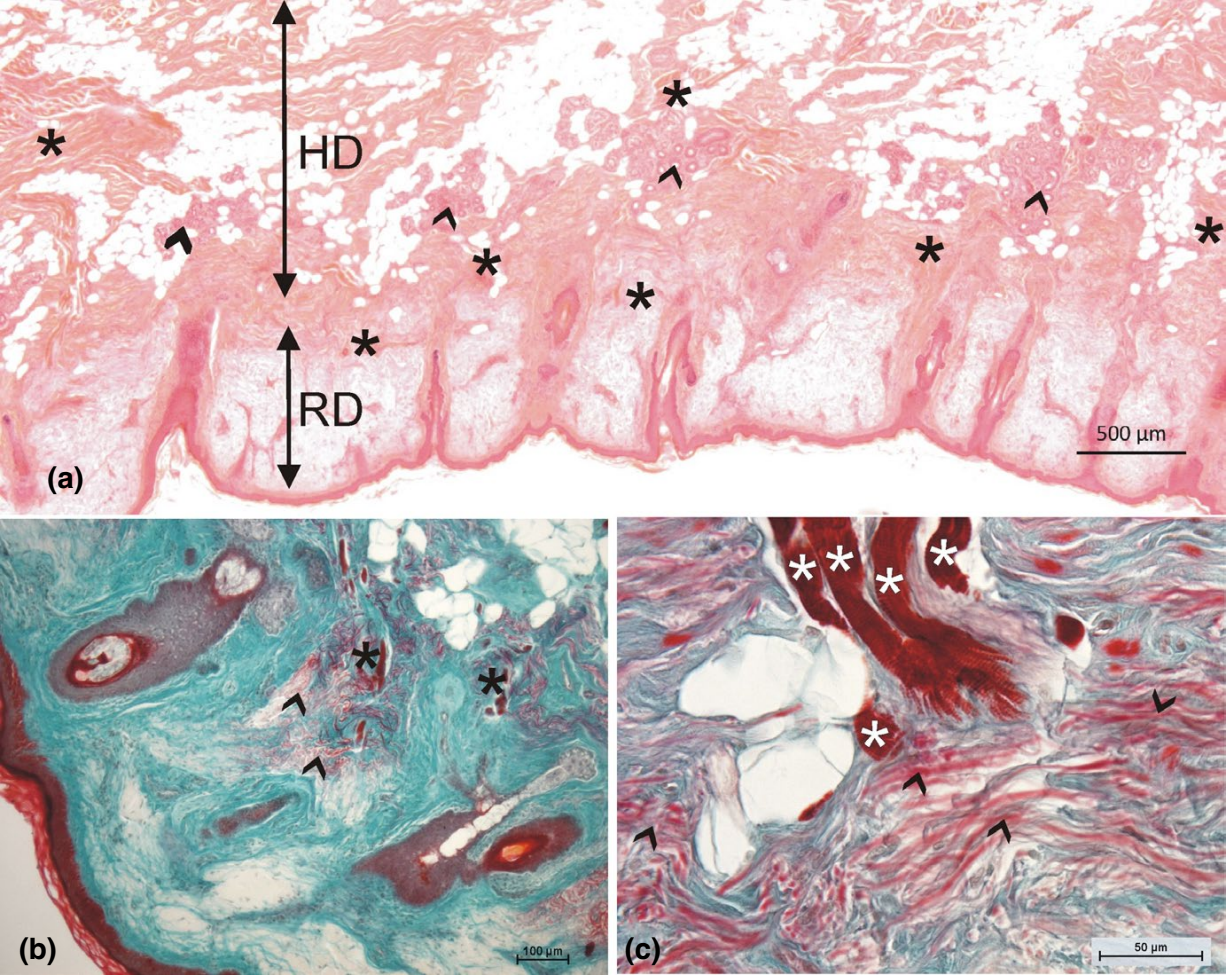
(a) Overview of the skin region next to the orbicularis oris muscle. Note the numerous muscle fiber bundles (asterisks) and sweat gland formations (arrowheads) in the hypodermis (HD). The reticular dermis (RD) appears pale. The thin papillar dermis follows the hair follicles. (b) Muscle fibers enter the dermis (asterisks) and end in regions with high amounts of elastic fibers (arrowheads). (c) Higher magnification shows the brush‐like arranged protrusions of the muscle fibers (asterisks); the elastic fibers (red, marked by arrowheads) are not parallel to the muscle fibers; in addition, the collagen fibers (green) are not densified in the regions of muscle fiber endings (a: HE staining; b,c: Goldner staining)

At certain places throughout the circumference of the orbicularis oris muscle, small groups of 5–15 striated muscle fibers branched off from the main muscle body, ran through the connective tissue strains of the subcutaneous layer (hypodermis), and reached the reticular dermis of the adjacent skin (Figure 2a). The skin architecture showed some specifics: the epidermal layer (mean thickness 41 ± 4 µm) was connected to the papillary dermis (mean thickness 31 ± 2 µm) which showed a flat appearance and followed the hair follicles toward the hypodermis. The reticular dermis (mean thickness 670 ± 85 µm) showed a resistance for staining resulting in a unique paleness (Figure 2a). In the inner third of the reticular dermis, the muscle fibers formed finger‐like protrusions which were brush‐like connected to the surrounding collagen tissue (Figure 2b). A closer investigation of this specific region revealed two different insertion zones: the most frequent one was a loosely arranged network of Type 1 collagen but a dominant presence of several layers of thick elastic fibers in the transition zone between the reticular dermis and the hypodermis, which were, however, not oriented toward the muscle endings (Figure 2c). Occasionally, the muscle fibers crossed the elastic‐rich inner part of the transition zone and inserted in the collagen network of the reticular dermis more superficially. There again, the collagen network was not oriented toward the muscle endings. The muscle fibers never reached the papillary dermis.

Neither the small bundles of striated muscle fibers nor the myocutaneous junctions showed any specific connection to the hair follicles or the sweat glands which were abundantly present in this skin region (Figure 2a). Sometimes, bundles of muscle fibers touched sweat gland formations but were never seen within this clew of glandular tissue. The myocutaneous junctions were strictly in the connective tissue of the reticular dermis and did not insert in the connective tissue surrounding the hair follicles and being continuous with the papillary dermis.

**FIGURE 3:**
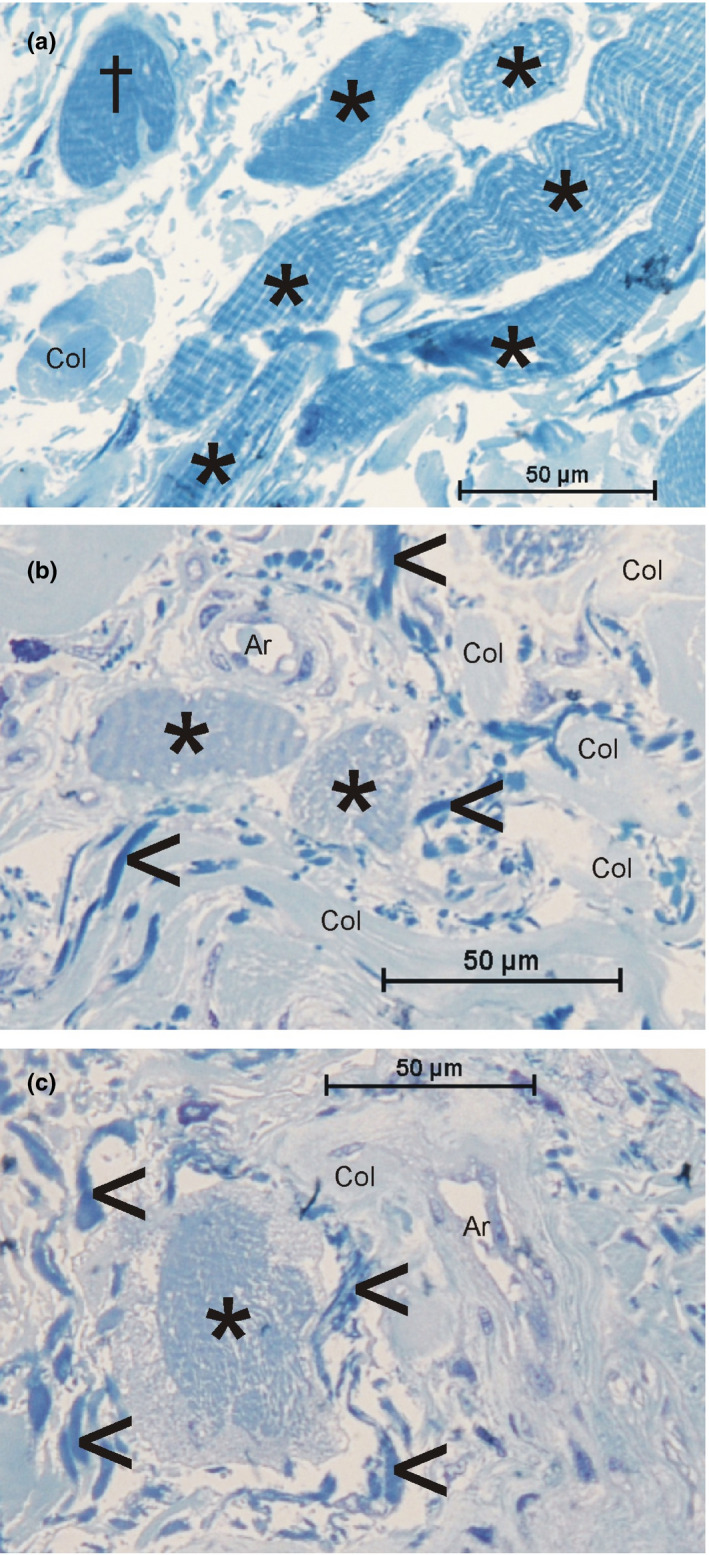
Toluidine blue‐stained semi‐thin sections of striated muscle fibers (asterisks) in the dermis (orbicularis oris muscle). (a) Bundle of muscle fibers; one fiber (cross) shows the beginning of finger‐like protrusions. (b,c) Single muscle fibers are surrounded by small vessels (Ar, arterioles) and elastic fibers (arrowheads). Col = bundles of collagen fibers

Semi‐thin sections revealed that the bundles of muscle fibers entering the dermis were often surrounded by a thin endomysial and distinct perimysial sheath containing of loosely arranged collagen fibers and mostly ground substance. Reaching toward their endings, the bundles lost their distinct sheath (Figure 3a) and the single fibers formed finger‐like protrusions containing myofilaments and showing basement membrane between them (Figure 3a). Single muscle fibers were often accompanied by small vessels (Figure 3b,c). Mainly, the bundles of collagen and the elastic fibers showed no specific arrangement toward the muscle fibers (Figure 3b). At places, however, a dense elastic network was seen around single muscle fibers just prior to their brush‐like ending (Figure 3c).

### 3.2 Collagen XXII

**FIGURE 4:**
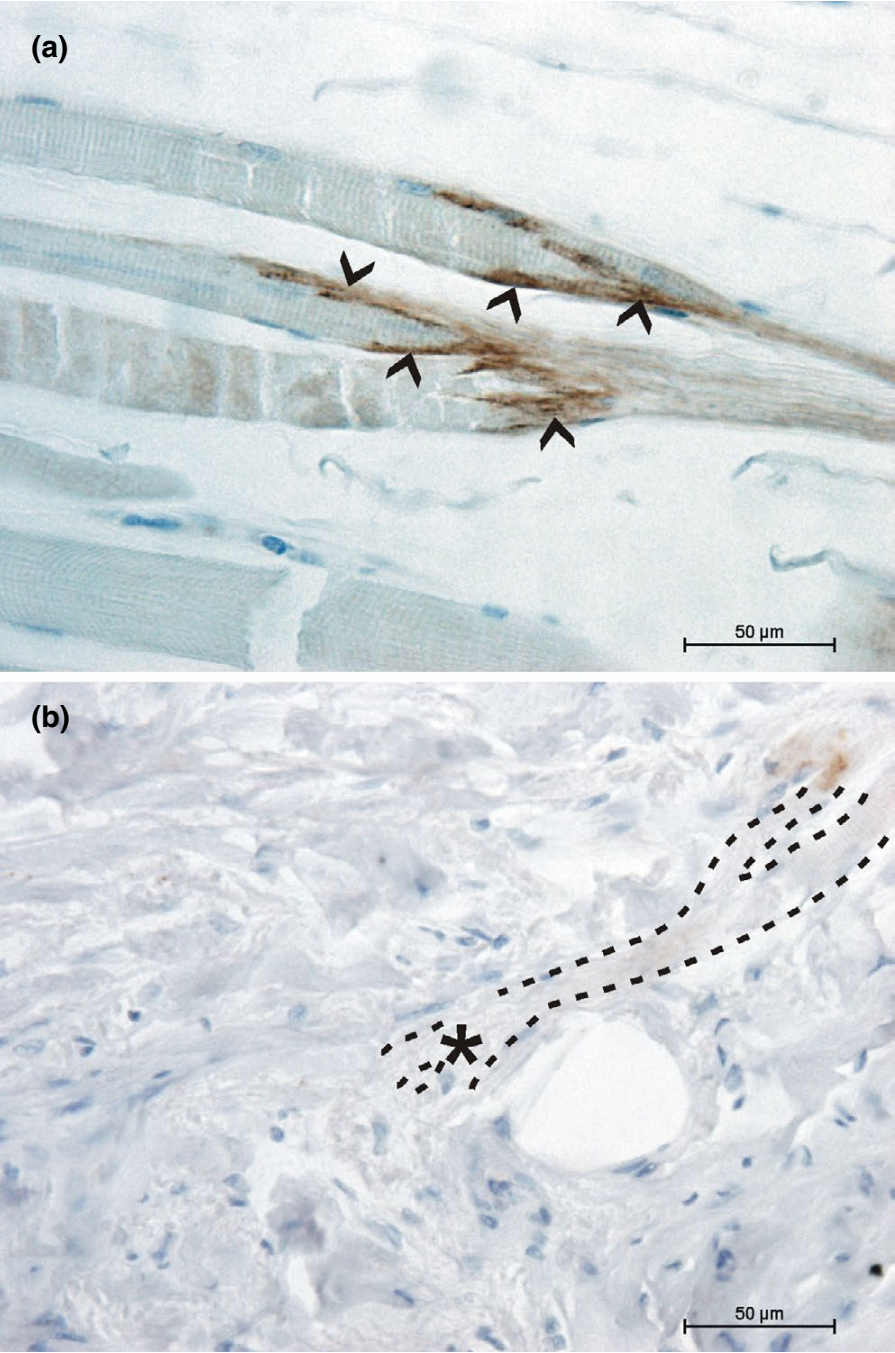
Collagen XXII at the ending of muscle fibers. (a) The myotendinous junction of the palmaris brevis muscle shows strong collagen XXII staining (arrowheads). (b) Muscle fibers of the orbicularis oris muscle reaching the dermal layer (highlighted with dotted lines) show no collagen XXII staining at their endings (asterisk)

At the myotendinous junction of the palmaris brevis muscle, intense staining of collagen XXII was present (Figure 4a). In contrast, the myocutaneous junctions of the orbicularis oris muscle showed no presence of collagen XXII at their interface (Figure 4b).

## 4 DISCUSSION

Summarizing our findings, true myocutaneous junctions exist in the human but are limited to the mimic muscles. Here, muscle fibers reach the dermis and insert in the fibro‐elastic network without locally modifying its three‐dimensional structure. This connection is different from myotendinous junctions and lacks collagen type XXII, a specific myotendinous junction protein.

Although it is a long known fact that the mimic muscles insert into the skin, not much attention was paid to a close morphology of this region. The term ‘myocutaneous junction’ in analogy to ‘myotendinous junction’ has not yet been established, although it seems appropriate since there are morphological differences between both junctions, for the first time described here. The limitation to facial mimic muscles, excluding the platysma, might underline the exclusiveness of this group of skeletal muscles including their lack of muscle‐specific sensory organs which are present in the platysma (May et al., 2018). It is tempting to speculate that the changes in muscle length usually sensed by muscle spindles in skeletal muscles can specifically be transduced by changes in skin tension to the facial mimic muscles via the myocutaneous interface superseding muscle spindles in this unique situation.

Surprisingly, the connective tissue of the dermis was not substantially modified by the insertion of the muscle fibers. More specific, the collagen and elastic components did not orient themselves toward the finger‐like protrusions of the muscle fibers which entered the network perpendicularly. The single muscle fibers seem not to create enough mechanical force to influence their surroundings (compare Huijing, 1999). This is in contrast to the suggested summation of several parallel muscle fibers at myotendinous or even myo‐myonal junctions (Zenker et al., 1990). This mechanical difference might also explain the lack of collagen type XXII at the myocutaneous junctions. An interesting observation noted only in the skin containing myocutaneous junctions was the virtually unstained reticular dermis. This observation is hitherto unmentioned in the literature and awaits further investigation.

Interestingly, not much attention was paid to the insertion of the panniculus carnosus/cutaneus trunci muscle in animals and its associated muscles in human. Here, we could show that muscles associated with the panniculus carnosus in man are more connected to the body fascia than to the dermis. This seems also true for the rat (Theriault & Diamond, 1988), although not morphologically demonstrated. Therefore, these muscles differ from mimic muscles showing at least in part true interaction with the skin dermal layers.

## AUTHOR CONTRIBUTIONS

CAM took tissue samples, performed the evaluation, and prepared the manuscript. SB performed the staining and reviewed the manuscript.

## Data Availability

All slides are available to view. Otherwise there is no statistic data block.
